# Pain Management in Cancer Patients Using a Mobile App: Study Design of a Randomized Controlled Trial

**DOI:** 10.2196/resprot.3957

**Published:** 2014-12-12

**Authors:** Stephen Agboola, Mihir Kamdar, Clare Flanagan, Meghan Searl, Lara Traeger, Joseph Kvedar, Kamal Jethwani

**Affiliations:** ^1^Center for Connected HealthBoston, MAUnited States; ^2^Massachusetts General HospitalBoston, MAUnited States; ^3^Harvard Medical SchoolBoston, MAUnited States

**Keywords:** cancer pain, mobile application, randomized controlled trial, self care, palliative care, mHealth

## Abstract

**Background:**

Despite the availability of effective medications and clinical guidelines for pain management, pain control is suboptimal in a sizeable proportion of patients with cancer pain. The National Comprehensive Cancer Network guidelines recommend a comprehensive and multimodal approach for management of cancer pain. We developed a mobile phone application, ePAL, based on clinical guidelines to empower patients for cancer pain management by prompting regular pain assessments and coaching for self-management.

**Objective:**

The objective of this study is to evaluate the effect of a multidimensional mobile phone-based pain management application, ePAL, on controlling cancer pain and improving quality of life in patients with cancer pain being treated at an academic palliative care clinic.

**Methods:**

The study will be implemented as a 2-arm randomized controlled trial with 110 adult patients with CP who own a mobile phone over a follow-up period of two months. Participants will be randomized to either the intervention group receiving ePAL and usual care or to a control group receiving only usual care. The brief pain inventory will be used to assess our primary outcome which is pain intensity. We will also evaluate the effect of the intervention on secondary outcomes which include the effect of the intervention on hospital utilization for pain crisis, quality of life, adherence to analgesic medications, barriers to pain control, anxiety and patient engagement. Instruments that will be used in evaluating secondary outcomes include the Brief Pain Inventory, Morisky Medication Adherence Scale, Barriers Questionnaire-II, Functional Assessment of Cancer Therapy–General, Edmonton Symptom Assessment System, Generalized Anxiety Disorder 7-item scale, and the Functional Assessment of Chronic Illness Therapy-Fatigue. The intention-to-treat approach will be used to evaluate outcomes. Our primary outcome, pain intensity, measured longitudinally over eight weeks, will be assessed by mixed model repeated analysis. Effect sizes will be calculated as mean group differences with standard deviations.

**Results:**

The study is still in progress. We hope to have results by the end of 2015.

**Conclusions:**

The multidimensional approach to pain management implemented on a mobile phone application could lead to significant improvements in patient outcomes.

**Trial Registration:**

ClinicalTrials.gov NCT02069743; https://clinicaltrials.gov/ct2/show/NCT02069743 (Archived by WebCite at http://www.webcitation.org/6Qb65XGGA).

##  Introduction

### Background

In addition to the inherent pathophysiological effects associated with any cancer, pain is one of the most feared consequences of cancer [1,2]. Cancer Pain (CP) may be present at any stage of the disease process. It may be the first complaint patients experience before diagnosis and may even be present long after treatment. Pain may be due to the direct effect of the cancer, diagnostic procedures or treatment. It is associated with distressing psychosocial discomforts and adversely impacts the patient’s quality of life [1]. The prevalence of CP varies depending on the type of cancer and stage of the disease; it ranges from 24%-60% in patients receiving treatment, 62%-86% in patients with advanced cancer, and 33% in patients post curative treatment [2-5]. Results from a meta-analysis showed that the prevalence of CP was 70%, 59%, 55% 54%, 52%, 60%, and 50% in head and neck, gastrointestinal, lung/bronchus, breast, urogenital, gynecological, and all cancer types respectively [6].

Despite the availability of guidelines from organizations such as the World Health Organization (WHO), at least 14% of patients with cancer pain have inadequate analgesia [7,8]. Barriers to the adequate management of CP may be physician or patient-related. Guidelines recommend that patients be screened (with standardized assessment scales) for CP at any encounter with their providers, but physicians generally under-assess pain, given their busy clinic schedules. Additionally, while some physicians have misconceptions about drug tolerance, addiction, routes of administration, and exaggerated fear of adverse effects that accounts for the sub-optimal management of CP [9]. Poor patient-physician communication is another reason for failure to accurately establish the intensity of the patient’s pain. In the United States, this is especially worse in ethnic minorities and contributes to further widening the health disparity gap [10,11]. Many patients want to be “good” patients and do not want to “trouble” their physicians. As a result they may have misconceptions about “pain as an unavoidable consequence of cancer”, fear of addiction, issues of medication compliance, and side effects from their pain medication [9]. These provider and patient-based barriers can culminate in a burden on the patient-provider relationship and result in inadequate treatment of cancer pain.

Cancer pain is multidimensional in nature and requires intensive self-management. It is important for affected patients to be coached, educated, and empowered to manage the pain they experience. Unfortunately, randomized clinical trials have been scarce on this subject [1]. The standard care in pain management is based on the analgesic ladder recommended by WHO from which most other guidelines have evolved. Although the guidelines have shown to be effective, evidence suggests that they are poorly implemented in practice [12]. Gaps in knowledge about pain, clinical applications of pain, and a perpetual disconnect between patient and provider regarding pain, are all impeding progress in developing best practices for CP management. The National Comprehensive Cancer Network (NCCN) guidelines recommend a comprehensive and multimodal approach for management of CP [3]. Pain can be controlled if recommended algorithms are systematically applied and tailored to meet patients’ needs [3].

Traditionally, management modalities are administered during visits to a clinician be it an oncologist, nurse or social worker. However, we developed a self-management mobile phone application, ePAL that patients can use in between visits to manage CP. The tool was developed based on clinical guidelines to empower patients for self-management by facilitating patient education, regular pain assessments, and timely feedback, medication changes and improving patient-physician connections. Our strategy is focused on supporting patients by regularly nudging them to assess their level of pain control and coaching them about CP management through regular messaging.

Our goal is to help patients with cancer pain gain better control of their symptoms and improve their overall quality of life. Therefore, we hypothesize that patients randomized to receive ePAL will have better pain outcomes, measured by pain intensity, and improved quality of life compared with patients receiving usual care.

### Specific Aims

#### Primary Aim

Our primary aim is to evaluate the effect of ePAL on pain control in patients with cancer pain.

#### Secondary Aims

Secondary outcomes to be assessed include evaluating the effects of ePAL on: (1) quality of life; (2) hospital utilization for pain crises; (3) adherence to analgesic medications; (4) daily opioid consumption; (5) levels of anxiety; and to evaluate (6) participants’ pattern of engagement with ePAL; and (7) perceived barriers to cancer pain management.

## Methods

### Trial Design

**Figure 1:**
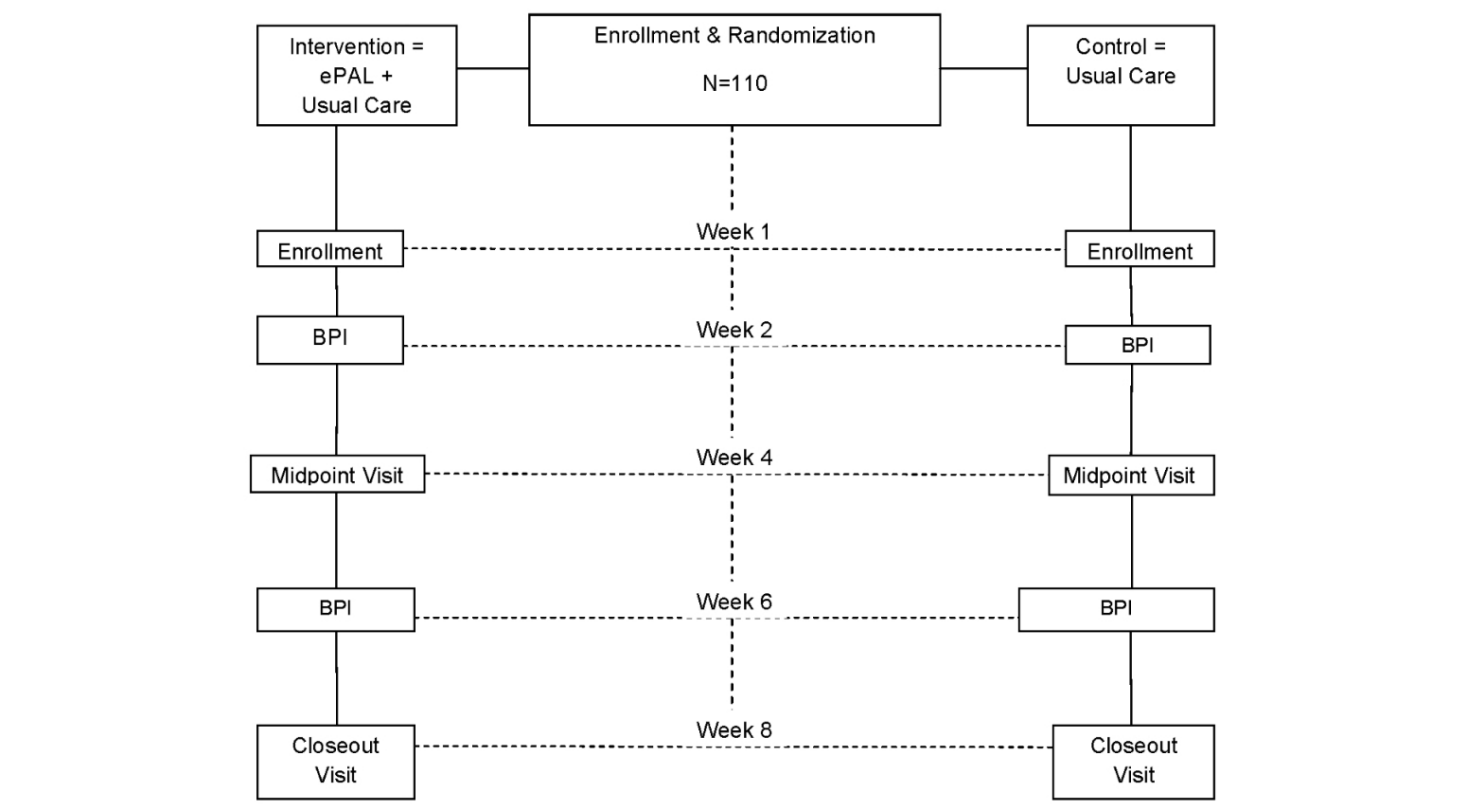
Schematic summary of the trial design. BPI: Pain intensity assessments with the Brief Pain Inventory.

This study will be implemented as a 2-arm randomized controlled trial (RCT; NCT02069743) with repeated assessments at baseline, midpoint, and at the end of the study over an eight weeks follow-up period. Figure 1 shows the research design.

### Participant Inclusion/Exclusion Criteria

Patients must meet all eligibility requirements to be enrolled into the study. Eligible patients are ambulatory, patients aged 18 years or older with diagnoses of a solid-organ cancer and moderate to severe levels (at least 4/10 on the numerical rating scale [NRS]) of pain at enrollment. They must also have a mobile phone and should be able to read and speak English. Ineligible patients will be defined as those who (1) have a life expectancy less than two months as determined by the clinical team; (2) have any significant medical or psychiatric comorbidities (other than depression or anxiety) or cognitive impediments that would prevent them from being able to utilize the program; (3) have a known history of substance abuse; and (4) participating in any other investigational therapies or other study protocols that may have an impact on pain intensity or quality of life which are the main outcomes of this study.

### Recruitment Procedure

All patients, regardless of ethnicity, with upcoming appointments at the Massachusetts General Hospital (MGH) Palliative Care Center are considered potential candidates. Therefore, potential subjects for this study will be drawn from adult cancer patients presenting with pain at the MGH Palliative Care Center. They will undergo screening to ensure eligibility and formal enrollment by signing the informed consent form. This is done before any study procedure is carried out. Participating subjects will complete all enrollment surveys and randomization procedures. All participants will be instructed to continue to receive medical care from physicians as usual. Subjects randomized to the intervention arm download the ePAL mobile application and will be taught how to use the functions of the application.

### Intervention

#### Framework for Intervention

##### Overview

The ePAL mobile application was designed to augment existing pain management protocols at the MGH Palliative Care Center and to promote self-efficacy for pain management in patients with cancer pain. Therefore, we use a comprehensive and multimodal approach as recommended by the NCCN guidelines for treatment of adults in addition to addressing common challenges in managing CP, as identified in oncologic clinical practice and in the medical literature [3]. The recommended targeted dimensions for CP management include providing support, encouraging subjects to report CP, teaching coping skills, and building self-efficacy. This app is based on the premise that providing a platform where patients can easily evaluate pain control and find support to manage their pain will overall improve pain management and quality of life. Therefore, ePAL (1) provides a medium for on-demand pain assessments using a NRS (ie, patients can use the app to assess the level of their pain control at any time when they experience exacerbation of symptoms or breakthrough pain); (2) coaches patients using bite-sized educational and supportive messages; (3) facilitates patient-provider communication; (4) helps patients track their symptoms, and (5) requests prescription refills. Furthermore, the intervention is tailored to the individual patient’s need which is determined by the ratings of pain intensity on the NRS, self-reported symptoms and self-reported barriers to pain management. These factors were taken into account in the design of the app and aided in determining the type of educational coaching messages that a particular subject receives. We further elaborate on our approach below.

##### Increasing Opportunities for Pain Control

Pain control is routinely assessed when a patient complains about it during a scheduled visit to the doctor’s office (every 2-6 weeks, depending on patient’s clinical condition) or during unscheduled patient-initiated visits for uncontrolled pain. ePAL on the other hand, can be easily administered outside of hospital settings. The app prompts participants to assess the level of their pain control over the past 24 hours three times per week. As a result, it provides patients with a medium to regularly evaluate pain control and opportunities for management. Participants with breakthrough pain or other CP-related complaints are able to perform ad hoc pain assessments and management at any time. The app recommends management strategies based on responses to the pain assessment and other pain management barriers. Treatment recommendations are not prescriptive but guide patients on what to do to help them manage their pain. Patients with newly occurring, persistent, or severe pain will be connected directly to the MGH Palliative Care Center for further evaluation and treatment. This is enabled by the automatic call function on the app that links directly to the MGH Palliative Care Center. However, patients have the option to decline speaking with the care team about the trigger symptom. For patients who need further evaluation outside of the regular pain clinic hours, they will be automatically connected with the Palliative Care on-call physician. In addition, participants can pursue medical assistance through the usual emergency phone and paging protocols at the Mass General Palliative Care Center and Cancer Center.

##### Coaching

Our hope is that by the time patients graduate from the program, at the end of the 2-month study, they will be better equipped to manage their CP in between their clinic visits or appropriately report uncontrolled pain. Contents from the educational library of ePAL will be used to coach participants on pharmacologic and nonpharmacological interventions to reduce CP. Coaching will also address the various misconceptions associated with CP management [12,13]. Patients are coached through small, bite-sized push notification with daily “tips” for managing cancer, cancer pain, and other side effects. These messages will help keep patients connected to their care providers even when they are not immediately due for another clinic visit, and facilitate improved communication between patients and providers. Additionally, coaching will also address medication adherence which will be encouraged by emphasizing the need for “by the clock” administration, addressing misconceptions about tolerance and addiction, managing side effects of analgesic therapy, and providing general education about cancer and CP [11-14].

#### Key Features of ePAL

##### Pain Assessments and Feedback

This is one of the key features of the app. Patients are nudged to assess level of their pain control three times per week. However, they are also able to perform ad hoc pain assessments apart from these scheduled prompts. Patients input the level of their pain control on a sliding NRS on the homepage of the ePAL. After patients rate their pain levels, the app will assess whether it is a new or recurring pain, the acceptability or tolerability of the pain, barriers to pain control and also rule out red flag symptoms (eg, persistent vomiting, no bowel movement for >3 days) that may require further evaluation by the care provider. Based on the identified barriers to adequate pain control, the app then suggests management steps and coaches them by addressing the identified barriers.

##### Multimedia Library

Patients often report a need for easily accessible and understandable information. For the ePAL mobile app, we developed a comprehensive library with information some of which were created by the investigators and others chosen from reliable sources. The library contains information on a wide array of topics relating to cancer, cancer pain, pain medication, side effects of pain medications, and other related symptoms, barriers to pain management, etc. The library is broken down into topic pages to facilitate users learning at their own pace instead of being overwhelmed by too much information. The app also includes videos (featuring physicians and nurse practitioners from the MGH Palliative Care Center explaining important topics in CP management) and audio files on relaxation and meditation techniques for pain management.

##### Notebook

The app offers a diary, directly accessible from the homepage and other relevant locations, for patients to log their experiences dealing with their symptoms and questions they have about their management protocols. This was incorporated into the app based on exploratory needs assessment where some patients reported having lots of questions for their providers which they forget to ask at the clinic visit and leave without hearing the answers. Patients can generate a report of all their notes and bring them up for discussion during the in-person visit. We hope the notes will help the patients better organize their discussions with their care providers during the clinic visits.

##### Tracking

The app tracks pain scores so that patients can monitor their progress and also share their trends with their care provider and support system. There are two available views: a sliding weekly scale and a monthly calendar view. Pain scores are displayed in green, yellow, and red which correspond to mild, moderate, and severe pain respectively. From here, users can also directly access notes associated with each day.

##### Medication Refill Request

This is an additional feature to bolster user engagement with the app and also to facilitate continuous pain management without the interruption of unfilled prescriptions. This is based on findings from exploratory needs assessment at the MGH Palliative Care Center where patients reported that calling the hospital for refills, pain consultations, appointment scheduling, etc could be stressful and time-consuming. Patients can request refills in one simple tap on the app.

**Figure 2:**
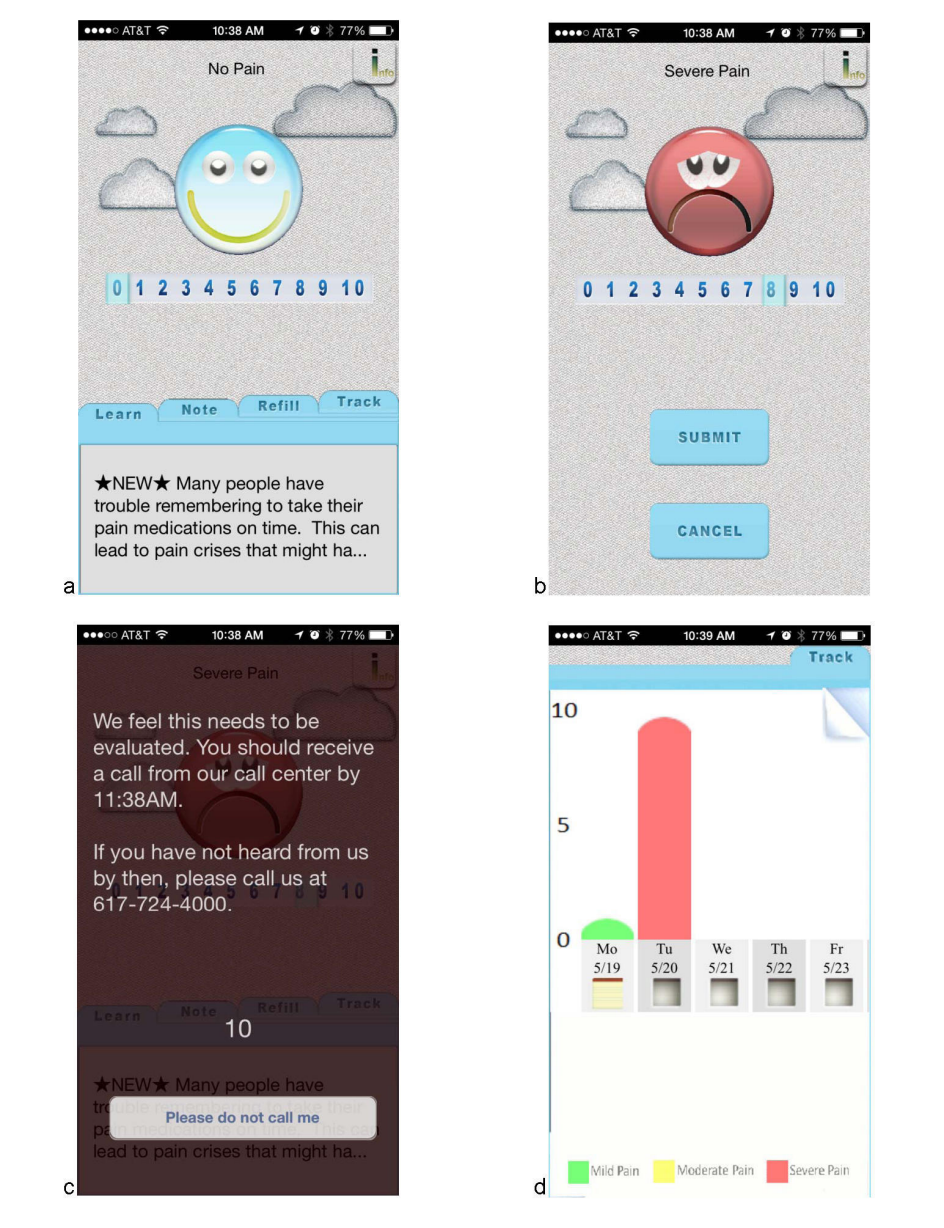
ePAL Mobile Application. a-d shows the numerical rating scale and pain tracking feature: a). Welcome page with educational tip; b & c). NRS and resulting message if pain score >8; d). Trend of pain scores, with notes.

**Figure 3:**
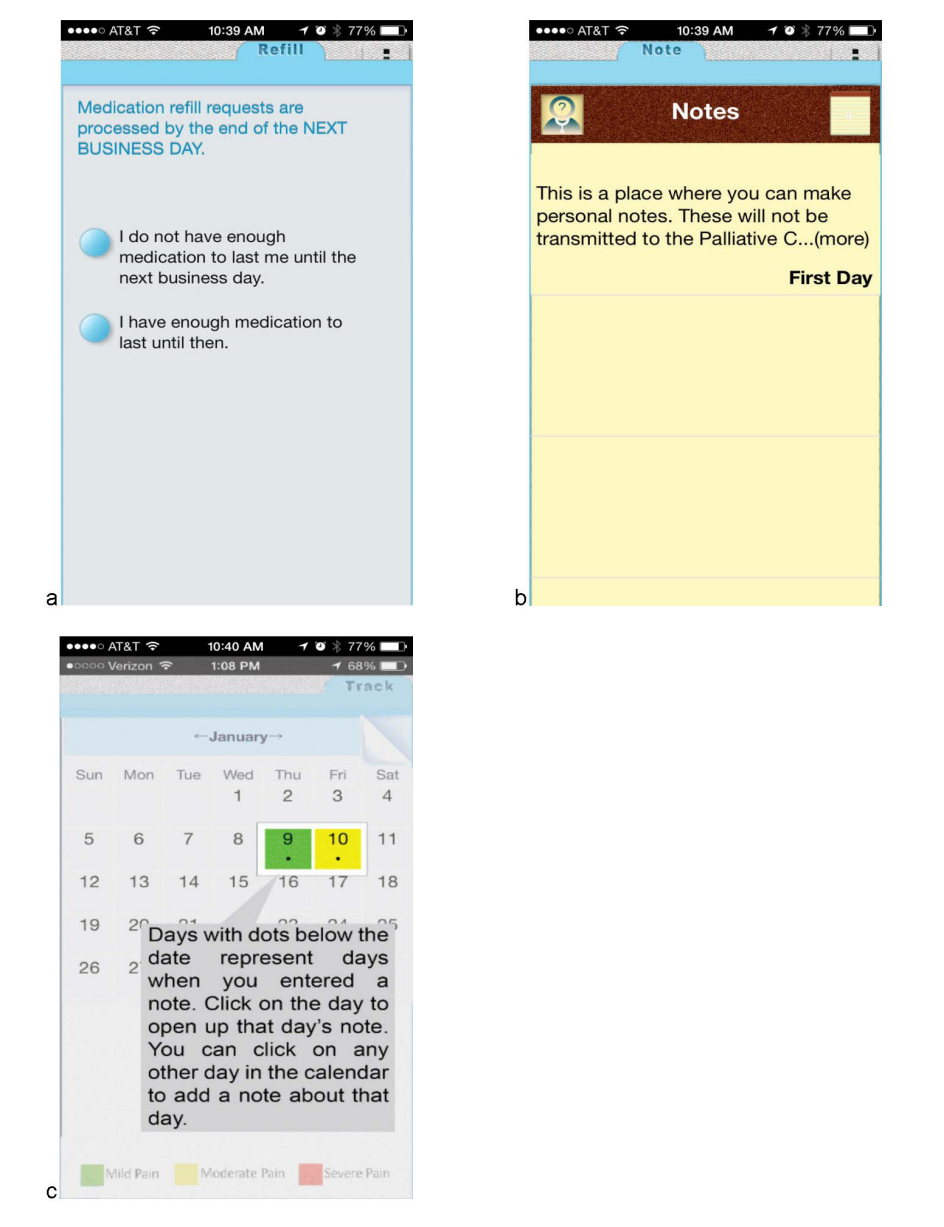
ePAL Mobile Application Features: a). Prescription refill; b). Notes feature; c). Calendar showing days with notes.

Screenshots of the ePAL mobile intervention are shown in Figures 2 and 3. Figure 2 shows the welcome screen of the application and a sample of educational messages (a), the resulting feedback from a pain score of >8 (b, c), and the symptom tracking function (d). In Figure 3, the additional features of the intervention are shown. These include the ability to order prescription refills and make notes for tracking and or with specific questions for the physician.

#### Intervention Group

Participants will continue to receive medical care from their oncologist, palliative care providers, primary care physicians and other care teams as usual. In addition to usual medical care, they will be able to use ePAL to manage their pain. During the study, intervention subjects will receive push notifications on their phones to assess their pain control over the past 24 hours every morning on Mondays, Wednesdays, and Fridays throughout the study period. The app will guide them through management steps based on their responses. Additionally, one coaching message will be sent to subjects once every day. Subjects will also be encouraged to use the app to manage their pain on-demand, and also to use other features of the app, including pain tracking, prescription refill requests, and the multimedia education library.

#### Control Group

Participants in the control group will continue to receive medical care from their care teams as usual. However, they will not be able to use the app during the study period.

### Outcome Measures

#### Data Collection Materials

There is one primary outcome and several secondary outcomes that will be assessed in this trial. This study uses several validated instruments for data collection: (1) the Brief Pain Inventory (BPI) assesses participants’ level of cancer pain and interference with daily function [15]; (2) the Morisky Medication Adherence Scale (MMAS-8) is an 8-item self-reported questionnaire used to measure adherence to medication [16]; (3) the Barriers Questionnaire-II (BQ-II) is used to capture subjects’ beliefs that may impact optimal pain control [17]; (4) the Patient-Health Questionnaire PHQ-8 is used to screen for depression [18]; (5) the Functional Assessment of Cancer Therapy-General (FACT-G) is used to measure health-related quality of life [19]; (6) Edmonton Symptom Assessment System (ESAS-r) is used to assess symptoms commonly found in palliative care patients [20]; (7) the generalized anxiety disorder 7-item scale (GAD-7) is an easy to use instrument to assess levels of anxiety [21]; (8) the Functional Assessment of Chronic Illness Therapy-Fatigue (FACIT-F) assesses levels of fatigue in patients with chronic illnesses [22].

#### Primary Outcome

Primary outcome measurement is pain intensity measured as a continuous outcome. It will be measured quantitatively with the BPI that assesses worst, least, average, and current pain intensity over the past week. The self-administered paper version will be used during in-person visits at baseline, midpoint, and at closeout; while the Interactive Voice Response (IVR) version will be used at weeks 2, 4, and 6.To minimize loss of data, any subject who is not able to complete the BPI at weeks 2 and 6 via IVR will be sent REDCap links to complete the surveys online. REDCap is a secure, Web-based application for data collection customizable for individual research studies. It is free and complies with all Health Insurance Portability and Accountability Act regulations (HIPAA). It was developed by a multi-institutional consortium initiated at Vanderbilt University.

#### Secondary Outcomes

All secondary outcomes will be assessed at midpoint and at the end of the study. Quality of life will be assessed with the FACT-G and ESAS-r. Hospital utilizations for pain crisis including emergency department or urgent care visits and unplanned clinic visits for pain will be assessed from the Electronic Medical Records. In addition, adherence to analgesic medications will be measured by the MMAS-8; opioid consumption will be measured quantitatively as oral morphine equivalent daily dose; patient-related barriers to pain will be measured quantitatively by the BQ-II; anxiety will be measured by the GAD-7; and pattern of patient engagement with ePAL will be assessed quantitatively by subject’s interaction with the system measured by the number of their responses to interactive messages and the use of other functions such as the notes function on the app.

#### Data Collection

Data will be collected at various time points (baseline, weeks 2, 4, 6 and 8) as described in Table 1. Data collection for weeks 2 and 6 will be via the IVR system while baseline, midpoint, and closeout visits will be conducted in-person. However, if for unavoidable reasons, in-person visits are not possible, survey questionnaires will be sent via REDCap. Outcome data will be stored in computer network files that are accessible only to IRB-approved study staff. Outcome data will be linked to each subject by a unique number, which will not have any identifying information. All data collected will be analyzed at the end of the study period.

**Table 1:** Data collection schedule: the table depicts the schedule for data collection.

Intervention/Control group data collection	At entry	2 weeks	4 weeks (+/- 5 days)	6 weeks	8 weeks Closeout (+/- 5days)
Enrollment questionnaire	X				
PHQ-8	X				
BPI	X	X	X	X	X
MMAS	X		X		X
BQ- II	X		X		X
FACT-G	X		X		X
ESAS-r	X		X		X
GAD-7	X		X		X
FACIT-F	X		X		X
Opioid consumption	X		X		X
Hospital utilizations					X

### Statistical Analysis Plan

#### Sample Size Estimation

A sample size of 88 subjects, 44 in each arm, is sufficient to detect a clinically important difference of 1.5 between the control and intervention arms in pain intensity scores assuming equal standard deviation of 2.5, using a two-tailed *t* test of difference between means, with 80% power and a 2-sided alpha of .05. Considering a dropout rate of 20%, the sample size required is 110 (55 per group). Fifty-five participants will be randomly assigned to the intervention and the remaining 55 participants will be assigned to the standard practice of care at the MGH Palliative Care Center (usual care group). Participants will be followed up for a total of 8 weeks.

#### Randomization

A computer program will be used to randomize subjects into the intervention or control arm in a ratio of 1:1 using random permutated blocks to optimize balance in each treatment at any given point in time during the study. Treatment assignment will be concealed in sealed envelopes prepared by a third party not directly involved with the study. Treatment assignment will be revealed after the consent form has been signed by the study participant. Due to the nature of this study, we will not be able to blind subjects to their treatment assignments. However, treatment assignments will be concealed from the investigators and the data analyst.

#### Statistical Analysis

Data analysis will be done with Data Analysis and Statistical Software: STATA, version 13 with an alpha of .05 set a priori for all analyses. All participants will be followed up for eight weeks and we will summarize their baseline demographic and technology use characteristics by study arm. Continuous variables will be compared between control and interventions using the *t* test and categorical variables will be compared using chi-square test. All analyses will be based on intention-to-treat in all randomized participants. Our primary outcome, pain intensity, measured longitudinally over eight weeks, will be assessed by mixed model analysis of variance with treatment assignment as between-group factor and time as within-subject factor. Effect sizes will be calculated as mean group differences with standard deviations. A similar approach will be used to analyze continuous secondary outcomes while categorical outcomes will be analyzed by chi-square test.

### Ethics and Informed Consent

Procedures of our methods have been reviewed and approved by the Dana-Farber/Harvard Cancer Center (DF/HCC) Institutional Review Board (IRB) and the study is registered at [23]. The app is secure and complies with all HIPAA requirements. Subjects will require a secure pass code to be able to access the app. All mobile phone numbers are stored in a secure shared drive available only to IRB-approved study staff. However, if any data breach or adverse effect occurs, the investigator will ensure that they are well-documented and reported according to the IRB’s requirements, regardless of causality.

Potential candidates with upcoming appointments at the Palliative Care Center will receive a letter informing them about the study. The study consent will also be sent along so that interested candidates can review and ask relevant questions during the enrollment visit. During the study visit, subjects will be given sufficient time to review the consent form and will be encouraged to ask questions. A research assistant will go through the consent form with subjects to ensure they comprehend details of the study. Thereafter, subjects will sign two copies of the consent form–one for them and the other for the study team. Patients who want more time to consider participating in the study will be sent home with an unsigned copy of the consent form and encouraged to think about it at their convenience. Should these patients choose to participate, they will be rescheduled for another enrollment visit. After the informed consent process, the research assistant will then perform all other enrollment procedures.

##  Discussion

### Principal Findings

Our expectation is that enabling patients to assess pain control on-demand and empowering them for self-management will lead to improvements in pain and quality of life outcomes. The ePAL mobile application enables patients to assess pain levels, track progression, manage cancer pain and associated symptoms outside hospital settings. Additionally, the app addresses patients’ beliefs that may hinder optimal pain control and also provides an easy way to connect with care providers for timely medication refills. When patients are educated and better understand their cancer and the pain that may be associated with it, improved skills on CP management can be adopted [12,14]. In addition to the capability to generate weekly symptom reports that could be shared with providers, the app also has a notebook function that allows patients to log their symptoms and other relevant questions to ask their care providers at their clinic visits. We hope these functions will facilitate improved patient-provider communication.

Cancer pain, when not managed timely or adequately, may progress to pain crisis necessitating immediate intervention. Pain crisis refers to an episode of severe, uncontrolled pain that causes patients and their caregivers significant distress [9,14]. Epidemiological data on incidence of pain crises in cancer management is scarce but some centers report that pain crises account for about 20-25% of all palliative care referrals [14]. Our intervention has the capacity to identify early on, cancer pain patients that are at risk of experiencing pain crises and eventual hospitalization.

A special concern is that the frequent prompts to assess pain control may cause a heightened awareness of symptoms and increase anxiety in some participants. While this is plausible, we hope that the application will increase self-efficacy for self-management. Therefore, the GAD-7 will be useful in establishing the effect of our approach on anxiety. A study by Kroenke and colleagues showed that a telecare management of pain and depression in patients with cancer actually had a positive effect on anxiety levels and was also associated with improvements in pain control and other quality of life outcomes in intervention participants [24]. Another similar concern is that the frequent pain assessments may also increase opioid use. Rustoen et al’s 6-week trial of PRO-SELF, a self-care intervention for cancer pain management that involved daily logging of pain intensity and analgesic intake, revealed that opioid intake increased significantly in all patients in the study [25]. They also reported that study participants took about 40% higher doses of opioids than reported in previous studies although they speculated that this could be due to the fact that only patients with bone metastases were included in the study [25]. It remains to be determined how ePAL will impact opioid consumption. However, this present study does not require daily logging of pain intensity but suggests pain assessments three times per week.

### Limitations

Given that the sample population is drawn from patients attending the palliative care clinic of a large academic center, majority of these patients may have advanced cancers and complicated diseases. As a result, there is a possibility that some patients may not be able to complete the study or attend final study visits. To limit loss to follow-up and missing data, we limit the participation to patients with life expectancy >2 months. Also, we will measure endpoints at multiple times and use REDCap for those unable to attend the final study visit. Another potential limitation is concerning the external validity of the study: This is because findings from this study may be generalizable to patients in academic medical centers. However, if the intervention proves to be effective in these settings, we believe that it will also be effective in patients receiving care in nonacademic medical centers because the app is patient-centered and easily adaptable to other settings.

### Conclusions

This is one of the first clinical trials evaluating the impact of a multidimensional evidence-based approach to pain management via a mobile phone application. Our hope is that findings from this study could lead to larger, multicenter trials to demonstrate more robust evidence. We also hope that similar evidence-based approaches can be developed for other acute-on-chronic conditions to empower patients for self-care, reduce cost, and improve overall patient outcomes.

## Abbreviations

CP: cancer pain
HIPAA: Health Insurance Portability and Accountability Act regulations
IRB: Institutional Review Board
IVR: Interactive Voice Response
MGH: Massachusetts General Hospital
NCCN: National Comprehensive Cancer Network
NRS: numerical rating scale
RCT: randomized controlled trial
WHO: World Health Organization
